# Human trafficking and the growing malady of disinformation

**DOI:** 10.3389/fpubh.2022.987159

**Published:** 2022-09-20

**Authors:** Jaya Prakash, Timothy B. Erickson, Hanni Stoklosa

**Affiliations:** ^1^Harvard Medical School, Boston, MA, United States; ^2^Division of Medical Toxicology, Department of Emergency Medicine, Mass General Brigham, Harvard Medical School, Harvard Humanitarian Initiative, Boston, MA, United States; ^3^Department of Emergency Medicine, Brigham and Women's Hospital and Harvard Medical School, HEAL Trafficking, Boston, MA, United States

**Keywords:** human trafficking, QAnon, infodemic, misinformation and disinformation, anti-trafficking activism, labor exploitation and human trafficking

## Abstract

Disinformation has endangered the most vulnerable communities within our world. The anti-trafficking movement in particular has been adversely impacted by disinformation tactics advanced through the QAnon campaign. QAnon's extremist messaging exacerbates gendered, racist, and xenophobic manifestations of trafficking victimization as well as problematic responses to trafficking that underpin historic structural inequities built into the United States' response to trafficking. We describe an overview of mechanisms used by the QAnon campaign to spread disinformation and illustrate how these mechanisms adversely affect the anti-trafficking movement. Given the critical role of healthcare providers in both the identification and connection to care for trafficked persons, as well as their susceptibility to disinformation, we provide several recommendations for the health sector to leverage their educational and advocacy power to combat trafficking disinformation while addressing the root causes of human trafficking.

## Introduction

Inaccurate information not only creates confusion but also endangers the most vulnerable communities globally. Complicating this threat is the role of social media, which has expedited and expanded the spread of information. The World Health Organization has recognized this as a public health issue and defined our current global state as an infodemic, or “too much information including false and misleading information” (1). Misinformation refers to fake or misleading information that is disseminated unintentionally. Disinformation refers to fake or misleading information that is intentionally disseminated (2).

Unfortunately, the anti-trafficking movement is no stranger to the role of misinformation and disinformation. Historical misconceptions have long underpinned public perception of human trafficking and continue to influence our policies, headlines, and biases. Inaccurate information exacerbates the disproportionate attention and resources dedicated to addressing the most sensational and “media-worthy” cases of human trafficking but overlook the spectrum of trafficking exploitation within the global landscape (3). Experts describe the deleterious impact this has on survivors of trafficking and increases marginalization of and lack of resources for migrant workers and those engaged in commercial sex 3. One particularly egregious example of this phenomenon are the conspiracy myths that lie at the foundation of the disinformation propagated by the QAnon campaign. Our goal within this manuscript is to (1) describe the large-scale mechanisms used by the QAnon campaign to spread disinformation, (2) illustrate how these mechanisms adversely affect the anti-trafficking movement on a population level, and (3) provide systems-level recommendations for the health sector to combat disinformation while addressing root causes of trafficking.

## The rise of QAnon

The QAnon campaign began in 2017 when an anonymous writer—under the name “Q”—began posting a series of coded messages on the website 4chan that implicated the involvement of “deep state” democrats, celebrities, media personnel, and political officials in a worldwide satanic domestic minor sex trafficking ring (4, 5). This then progressed to multiple anonymous imageboard sites by the names of 4chan, 8chan, and 8kun also weaponizing these messages to recruit following for conspiracy theories (6). Studies have found that accessibility of the internet, availability of social media, and abundant disinformation sources all catalyzed the grown of the QAnon campaign to an unprecedented threat level (7). With the unveiling of Jeffrey Epstein's involvement in the commercial sex industry, these conspiracy theories began infiltrating mainstream social media as well as public discourse. It migrated and integrated into larger online platforms such as YouTube and Reddit. Once efforts began mobilizing off-line, these theories were picked up via news coverage, which then broadened the message even further (8). Today, “QAnon has transformed from a bizarre conspiracy theory circulating only in the dark corners of cyberspace to a mainstream phenomenon advocated by politicians and media figure alike” (6, 9–11). Mobilization of this campaign has launched hundreds of #SaveTheChildren rallies across the United States (4). By late 2020, nearly 50% of Americans have heard QAnon conspiracy theories with surveys showing almost 14% of polled Americans identify themselves as “QAnon believers” (5, 12).

## Themes of disinformation

QAnon's large-scale beliefs are rooted in extremism identifying one absolute enemy, typically individuals with elite power, driving domestic minor sex trafficking (13). As is the case for most extremist theories, radicalization “discourages the navigation of differing perspectives required in a pluralistic society, and the dehumanizing effects of conspiracy which are important for supporting radicalization” (14). In the case of QAnons's campaign within the context of the anti-trafficking movement, their sensationalized theories around domestic minor sex trafficking propagate a simplified representation of exploitation that distracts energy and resources from the multi-systemic causes that ultimately give rise to human trafficking.

QAnon mobilizes gendered, racist, and xenophobic manifestations of trafficking victimization as well as problematic heroism responses to trafficking. “The ‘white slave' panic in Europe and the United States at the turn of the twentieth century” was driven by “those who fomented the white slavery scare of the time” and “sought to expose precisely the mobile yet highly organized net of the underworld lurking below the surface of society” (15). This is replicated in QAnon's panic regarding “the procurement, by force, deceit, or drugs, of a white woman or girl against her will, for prostitution” (15). The ways in which QAnon profiles victims of human trafficking simultaneously strengthens the conflation of human trafficking with exclusively sex trafficking despite other forms of trafficking exploitation having been found more prevalent globally. This systemic devaluing of trafficked individuals ignored by QAnon's profiling—people of color, immigrants, people who identify as gender non-binary—underpins historic structural inequities that have been built into the United States' response to human trafficking (16). At the core of both, we see “racialized and gendered roles of feminine helplessness and protection by traditional (i.e., masculine) institutions” (14).

QAnon's public messaging carries adverse implications for survivors and the anti-trafficking movement. Resources invested in inaccurate information divert resources and effort from supporting victims and survivors (17). In fact, QAnon's campaign could threaten the bipartisan support extended to this issue, driving potentially ineffective and dangerous policies based upon inaccurate information. By invalidating the narratives of people with lived experience and drowning out the voices of survivor-advocates, victims themselves may be less likely to self-report (17). Service providers will also be impacted through QAnon's efforts that perpetuate systemic biases and ultimately reinforce inequitable funding cycles, screening efforts, and care coordination.

## Mechanisms of disinformation

### False storytelling

Embedded within QAnon's online and offline disinformation are narratives of stolen innocence and virtue that position trafficked persons into victims with feminine identities centered around purity, innocence, and motherhood (18). They sensationalize embodiment of storytelling that is accepted and propagated by media coverage (19). This narrative of a heroic spectator with the potential to save the pure victim from the omnipotent evil clutches of human trafficking normalize problematic dynamics: “traditional power relationships, the construction of trafficking as a cudgel against the other, and the co-optation of discourse by institutional power” (14). In fact, QAnon-related images have been shown to markedly over-represent preteen white children when compared to the evidence on child trafficking demographics and also draw significantly upon graphic imagery that relies on the motif of distressed children (20).

Story-telling is particularly useful for disinformation because narratives are positioned to not only convey information but also serve as political instruments for spreading disinformation concerning human trafficking. First, if a story is of high quality, the events and lessons of the story are often perceived by audiences to provide a sound message and speak to a higher point. The power behind these stories then lies within their capacity to remind us of other compelling stories and in turn blurs the line between history vs. memory (14). Second, stories can draw upon larger social structures such as the sincerity of a storyteller or norms around performance that influence social acceptance of the narrative itself into our collective identity (19, 21). These characteristics of narratives that aim to (1) facilitate connections between people as well as ideas and (2) generate meaning from scattered pieces of information can be leveraged to spread disinformation about human trafficking (19, 21). This second point is particularly true within the context of the QAnon campaign given the finding that people are more likely to joining radicalized groups if they feel uncertain, fearful, or powerless within the context of their own lives (22). For audiences who are bombarded by a flood of information and without bandwidth to evaluate this information it seems that the various shortcuts and connections presented by distorted human trafficking narratives are used to process information (23).

### Co-opting

Extremist groups, such as the QAnon movement, also have the tendency to latch onto issues of national concern to promote their disinformation efforts, drawing attention to their conspiracies by repackaging reactionary views into messaging “save the children” and “stopping the traffickers” (14, 24). In fact, analysis of claimed motivations put forth by the #SaveTheChildren rallies “highlights the utility of moral-high ground arguments as a useful trope for advancing other motivations, often individual or political…An overarching commitment to “ending” trafficking or “saving” children thus emerged as a uniting theme, bringing together differing communities with different underlying or secondary motivations for joining the movement” (25). In fact, social media content analysis of QAnon users are notable for holding simplistic mental models regarding the battle between “good” vs. “evil” (26, 27).

With that said, through co-opting mechanisms, QAnon invalidates the lived and professional contributions made by survivors of labor and sex trafficking. It tokenizes and sensationalizes real trauma while simultaneously appropriating the anti-trafficking movement for political incentives (28). Disinformation spread in this manner additionally presents changes for anti-trafficking educators who must respond to these efforts without providing conspiracies a larger platform and more legitimacy (14, 29). Unfortunately, there is a wealth of evidence in rumor psychology to support that increased exposure afforded to disinformation, even if successful in discrediting, can mean inaccurate beliefs “still tend to persist, but in a weakened state” (29). In other words, so long as the QAnon beliefs or conspiracies are commented upon, they have the potential to gain traction and be integrated within the audience's mental models regarding human trafficking as well as our sociopolitical climate (30).

### Echo chambers and filter bubbles

Echo chambers and filter bubbles within the internet as well as social media can further concentrate the spread of disinformation. Echo chambers are communities of individuals who are consistently exposed to conforming opinions (31). Filter bubbles or ideological frames are states of intellectual isolation that result from personalized searches when website algorithms can selectively predict what information an individual user prefers, based on previous computer click-behavior or search histories (31). Regarding the phenomenon of both, a prior line of literature has demonstrated that the role of increased exposure to inaccurate, unsubstantiated information is associated with increased tendency of users to believe the content (32). Echo chambers, for example, are thought to be applied by information users to “process information through a shared system of meaning and trigger collective framing of narratives that are often biased toward self-confirmation” (33). Analysis of over 800,000 tweets regarding QAnon from the summer of 2018 found that the majority of users were disseminating rather than producing information, which created and sustained online echo chambers (26).

These findings demonstrate how people connected to QAnon supporters or beliefs are more likely to find and share content that supports similar perceived narratives of human trafficking and disregard any conflicting information (33). In fact, a prior randomized experiment by Kim and Cao (34) demonstrated how messaging and conspiracy can breed “a spiral of distrust; that is, exposure to the content leads to belief in conspiracies that causes heightened distrust, and the heightened distrust, in turn, makes people more susceptible to the influence of the content, which further increases distrust.” This social reinforcement strengthens polarization, support, and dissemination of these conspiracy theories. Although they are likely to assimilate more slowly into audience's perception of human trafficking, they are positively correlated with longevity of the belief and degree of sharing (33).

## Recommendations for the health sector

Healthcare providers are key actors in the anti-trafficking response for both identification of trafficked persons as well as connections to care. With that said, providers are also exposed and susceptible to the disinformation tactics employed within the current sociopolitical landscape. There are several ways health systems and professionals can leverage their educational and advocacy power to combat trafficking disinformation and invest in addressing the root causes of human trafficking (35).

First, it is vital that those with lived experience, from a diversity of demographics, shape health sector efforts on trafficking. Clinician educators should avoid sources that incorporate sensationalized language within discourse regarding human trafficking (e.g., avoid imagery of victim in restraints). Information alleging complex conspiracy theories without supporting evidence should be avoided. Medical educators must also be cautioned against using information disseminated by organizations that (1) promote racial, ethnic, or religious stereotypes and (2) claim to “rescue” trafficked “victims.” Health professionals should be trained on the diversity of people who experience trafficking, especially counter-stereotypical examples of trafficking, in order to combat structural and institutional biases that impact identification and intervention efforts for this population. Further, rather than “screening for trafficking,” providers can educate their patients about their rights as workers and connect them to resources because many victims may not self-identify (35).

Health systems should develop policies that identify and respond to individuals who have experienced any form of human trafficking, inclusive of labor and sex trafficking. They can disseminate accurate information about trafficking exploitation and worker rights to counter disinformation. Health professionals and health systems can advocate for policies that address social determinants of health that are also systemic vulnerabilities to human trafficking such as access to housing, social, legal, and/or employment support. Health systems should also advocate for an end to discriminatory practices against immigrants and communities of color.

## Data availability statement

The original contributions presented in the study are included in the article/supplementary material, further inquiries can be directed to the corresponding author/s.

## Author contributions

JP: investigation, methodology, writing—original draft, and writing—review and editing. HS: conceptualization, investigation, methodology, resources, supervision, validation, and writing—review and editing. TE: conceptualization, resources, supervision, validation, and writing—review and editing. All authors contributed to the article and approved the submitted version.

## Funding

This work was funded by NIH NIDA SBIR R44DA051106 and R44DA051106-02S1.

## Conflict of interest

The authors declare that the research was conducted in the absence of any commercial or financial relationships that could be construed as a potential conflict of interest.

## Publisher's note

All claims expressed in this article are solely those of the authors and do not necessarily represent those of their affiliated organizations, or those of the publisher, the editors and the reviewers. Any product that may be evaluated in this article, or claim that may be made by its manufacturer, is not guaranteed or endorsed by the publisher.
